# The Typhoid Toxin Promotes Host Survival and the Establishment of a Persistent Asymptomatic Infection

**DOI:** 10.1371/journal.ppat.1005528

**Published:** 2016-04-07

**Authors:** Lisa Del Bel Belluz, Riccardo Guidi, Ioannis S. Pateras, Laura Levi, Boris Mihaljevic, Syed Fazle Rouf, Marie Wrande, Marco Candela, Silvia Turroni, Claudia Nastasi, Clarissa Consolandi, Clelia Peano, Toma Tebaldi, Gabriella Viero, Vassilis G. Gorgoulis, Thorbjørn Krejsgaard, Mikael Rhen, Teresa Frisan

**Affiliations:** 1 Department of Cell and Molecular Biology, Karolinska Institutet, Stockholm, Sweden; 2 Department of Histology and Embryology, School of Medicine, University of Athens, Athens, Greece; 3 Department of Microbiology, Tumor and Cell Biology, Karolinska Institutet, Stockholm, Sweden; 4 Department of Pharmacy and Biotechnology, University of Bologna, Bologna, Italy; 5 Department of Immunology and Microbiology, University of Copenhagen, Copenhagen, Denmark; 6 Institute of Biomedical Technologies, Italian National Research Council, Segrate, Milan, Italy; 7 Centre for Integrative Biology University of Trento, Trento, Italy; 8 Institute of Biophysics of CNR, Trento, Italy; 9 Biomedical Research Foundation, Academy of Athens, Athens, Greece; 10 Institute for Cancer Sciences, University of Manchester, Manchester Academic Health Science Centre, Manchester, United Kingdom; 11 Manchester Centre for Cellular Metabolism, University of Manchester, Manchester Academic Health Science Centre, Manchester, United Kingdom; University of California Davis School of Medicine, UNITED STATES

## Abstract

Bacterial genotoxins, produced by several Gram-negative bacteria, induce DNA damage in the target cells. While the responses induced in the host cells have been extensively studied *in vitro*, the role of these effectors during the course of infection remains poorly characterized. To address this issue, we assessed the effects of the *Salmonella enterica* genotoxin, known as typhoid toxin, in *in vivo* models of murine infection. Immunocompetent mice were infected with isogenic *S*. *enterica*, serovar Typhimurium (*S*. Typhimurium) strains, encoding either a functional or an inactive typhoid toxin. The presence of the genotoxic subunit was detected 10 days post-infection in the liver of infected mice. Unexpectedly, its expression promoted the survival of the host, and was associated with a significant reduction of severe enteritis in the early phases of infection. Immunohistochemical and transcriptomic analysis confirmed the toxin-mediated suppression of the intestinal inflammatory response. The presence of a functional typhoid toxin further induced an increased frequency of asymptomatic carriers. Our data indicate that the typhoid toxin DNA damaging activity increases host survival and favours long-term colonization, highlighting a complex cross-talk between infection, DNA damage response and host immune response. These findings may contribute to understand why such effectors have been evolutionary conserved and horizontally transferred among Gram-negative bacteria.

## Author Summary

Bacterial genotoxins cause DNA damage in the host cells, resulting in activation of the classical DNA damage response, similarly to other well-characterized genotoxic agents. Three types of bacterial genotoxins have been identified: the cytolethal distending toxin (CDTs) family, the typhoid toxin and the peptide-polyketide colibactin, all produced by Gram-negative bacteria. The presence of genetic mobile elements upstream and downstream of the genes encoding for some CDT members suggests horizontal transfer, and indicates that these effectors may play a key function in bacterial infection/colonization. However, it is not clear whether these effectors play a role as virulence factors. In this work, we have addressed the effect of one of these genotoxins, the typhoid toxin, in the context of a natural infection. We used *Salmonella* Typhimurium, a bacterium that can establish persistent asymptomatic infections in immunocompetent individuals. We provide evidence that the presence of the functional genotoxin promotes the survival of the infected hosts and favours long-term asymptomatic colonization. Our work contributes to highlight the role of these microbial toxins in the complex interplay between the host and pathogenic bacteria, and poses a semantic and biological question on whether these effectors can be defined as “toxins”.

## Introduction

Genotoxins have been recently identified as a novel family of microbial effectors in pathogenic and commensal bacteria [1]. At present, three types of bacterial genotoxins have been identified. Two are protein toxins: the cytolethal distending toxin (CDT) family, produced by Gram-negative extracellular pathogens, such as *Escherichia coli*, *Aggregatibacter actinomycetemcomitans*, *Haemophilus ducreyi*, *Campylobacter* sp. and *Helicobacter* sp. (reviewed in [2]), and the typhoid toxin produced by the facultative intracellular pathogen *Salmonella enterica* serovar Typhi (*S*. Typhi) [3]. The third known genotoxin, colibactin, is a peptide-polyketide produced by strains belonging to the phylogenetic group B2 of *E*. *coli* (reviewed in [1]).

CDTs are AB_2_ toxins [4] and the typhoid toxin is an A_2_B_5_ toxin [5], where “A” stands for active subunit and “B” for binding moiety. The two toxins share a common feature, namely the presence of the CdtB, the “A” subunit, which is structurally and functionally homologous to mammalian DNase I. The additional active subunit in the typhoid toxin is homologous to the A subunit of the pertussis toxin, and possesses an ADP-ribosyl transferase activity, for which the cellular targets have not been yet identified [5]. Binding to target cells, and possibly also internalization, of the DNase-like subunit of CDTs and the typhoid toxin are mediated by the “B” subunits CdtA/CdtC and PltB, respectively [4,5].

The cellular responses to CDT, and to a lesser extent to the typhoid toxin, have been studied in *in vitro* models [1,6]. Intoxication causes the formation of DNA strand breaks in target cells, and activates the classical DNA damage response (DDR) orchestrated by the phosphatidylinositol 3-kinase-like protein kinase ataxia telangiectasia-mutated (ATM) [7–18]. As a consequence of the DDR activation, cells are arrested in the G1 and/or G2 phases of the cell cycle. Failure to repair the damage induces senescence or apoptosis in a cell type-dependent manner [2,19]. Intoxicated cells that survive and overcome the DDR-induced cell death or cellular senescence accumulate genomic instability and acquire the capacity to grow in an anchorage independent manner [20], two of the hallmarks of cancer [21].

To what extent bacterial genotoxins act as *de facto* virulence factors during *in vivo* infections is less clear. Furthermore, it is not fully understood whether the carcinogenic effect described in *in vitro* models would also be relevant in the context of chronic infections. This issue is highly relevant because persistent asymptomatic infections with *S*. Typhi [22] are associated with increased risk for tumor development in humans [23–25]. Several lines of evidence indicate that the presence of CDT promotes gastric or intestinal colonization in mouse models of infections with *C*. *jejuni* [26,27] or *H*. *hepaticus* [28,29]. These conditions are associated with enhanced inflammatory response in the gastric or intestinal mucosa [28,30,31], and development of hepatic dysplastic nodules 10 months after infection [32]. Studying the effect of the typhoid toxin *in vivo* poses a challenge, since this bacterium is a strict human pathogen. Song *et al*. demonstrated that intraperitoneal injection of a mutant *S*. Typhi strain carrying a deletion of the PltB subunit exhibits an increased ability to replicate in the liver and spleen of immunodeficient Rag2^-/-^ γc^-/-^ humanized mice [33], while purified typhoid toxin injected intravenously recapitulates the effect of typhoid fever in C57BL/6 mice [5]. It is noteworthy that most of these *in vivo* studies have been performed either in immunodeficient mice [26–28,30,31], or using non-physiological routes of infection [5,33]. To overcome these limitations, we developed two *Salmonella enterica* serovar Typhimurium (*S*. Typhimurium) strains, which cause systemic typhoid fever-like infection in immunocompetent mice and that, in contrast to ordinary S. Typhimurium, express either an active or inactive typhoid toxin [3,18,34,35]. This model provides a useful tool to study the role of the typhoid toxin in chronic infection and carcinogenesis in the context of a natural route of infection in immunocompetent subjects.

We show that under these conditions, the presence of the typhoid toxin promotes the survival of the host and favours the establishment of the status of a persistent asymptomatic carrier. Furthermore, we provide evidence that these effects are associated with modulation of the host immunoresponse and the intestinal microbiota.

Our data collectively highlight a novel aspect of typhoid toxin as an immune modulator, which reduces the intestinal inflammatory response and the clearance of the bacteria.

## Results

### Infection with the toxigenic *S*. Typhimurium strain enhances host survival

Studying the effect of the typhoid toxin in the context of a murine model is challenging since *S*. Typhi is strictly human-adapted [34]. In contrast, selected non-typhoidal serovars, such as *S*. Typhimurium, cause a disease in mice that resembles human typhoid fever [34,35], but do not harbor the toxin [3,18]. To overcome this difficulty, we cloned the *S*. Typhi *pltB-pltA* and *cdtB* genes [3,18] under the control of their endogenous promoters (S1A Fig), and transferred them by homologous recombination into the genomic *proV* gene of the fully virulent *S*. Typhimurium MC1 strain and the isogenic attenuated MC71 strain that carries a single point mutation within the gene coding for polynucleotide phosphorylase (*PNPase*). The latter can establish a persistent infection in BALB/c mice [35], which are highly susceptible to *S*. *enterica* infection due to a polymorphism of the *Nramp1* gene [36]. These strains (respectively designated MC1-TT and MC71-TT) are hereby designated as toxigenic strains. As a control, we constructed two isogenic strains carrying a non-functional genotoxin, due to the deletion of the *cdtB* gene (respectively designated MC1-Δ*cdtB* and MC71-Δ*cdtB*). Each typhoid toxin subunit was engineered with a unique C-terminal epitope-tag to allow detection of the protein in western blot or immunohistochemical analysis (S1A Fig).

The MC1 recombinant strain carrying the active toxin showed levels of entry and replication in epithelial cells comparable to those observed for the MC1*-ΔcdtB* (S1B Fig) indicating that expression of the active genotoxin does not alter the invasive capacity. The toxin genes encoded by *S*. Typhi are expressed only when the bacterium is internalized by the host cells and replicates within the *Salmonella* containing vacuole (SCV) [3]. To assess whether a similar regulation is also present in the *S*. Typhimurium strains, MC1-TT and MC1-Δ*cdtB* were grown for 24h in LB medium or in minimal medium pH5.8 (MM5.8) that mimics the condition of the SCV [37], and protein expression was visualized by western-blot (S1C Fig). The typhoid toxin subunits were strongly expressed when bacteria were cultured in MM5.8 medium, while minimal or no protein expression was observed in bacteria grown in LB medium, indicating that the regulation of the gene expression in the recombinant strains recapitulates that observed in *S*. Typhi. Similar results were obtained for the MC71 strains (S1C Fig). A last set of experiments was performed to investigate the ability of the toxigenic strain to induce DNA damage. CaCo-2 cells infected with MC1-TT exhibited high levels of DNA damage 24h post-infection, assessed by phosphorylation of the histone H2AX (γH2AX), while no specific γH2AX signal was observed in cells infected with the control MC1-Δ*cdtB* strain (S1D Fig). Similar results were obtained for the MC71 strains (S1D Fig).

These data demonstrate that the recombinant strains recapitulate the expression pattern and activity of the *S*. Typhi toxin, validating the bacterial strains used in this study.

**Fig 1:**
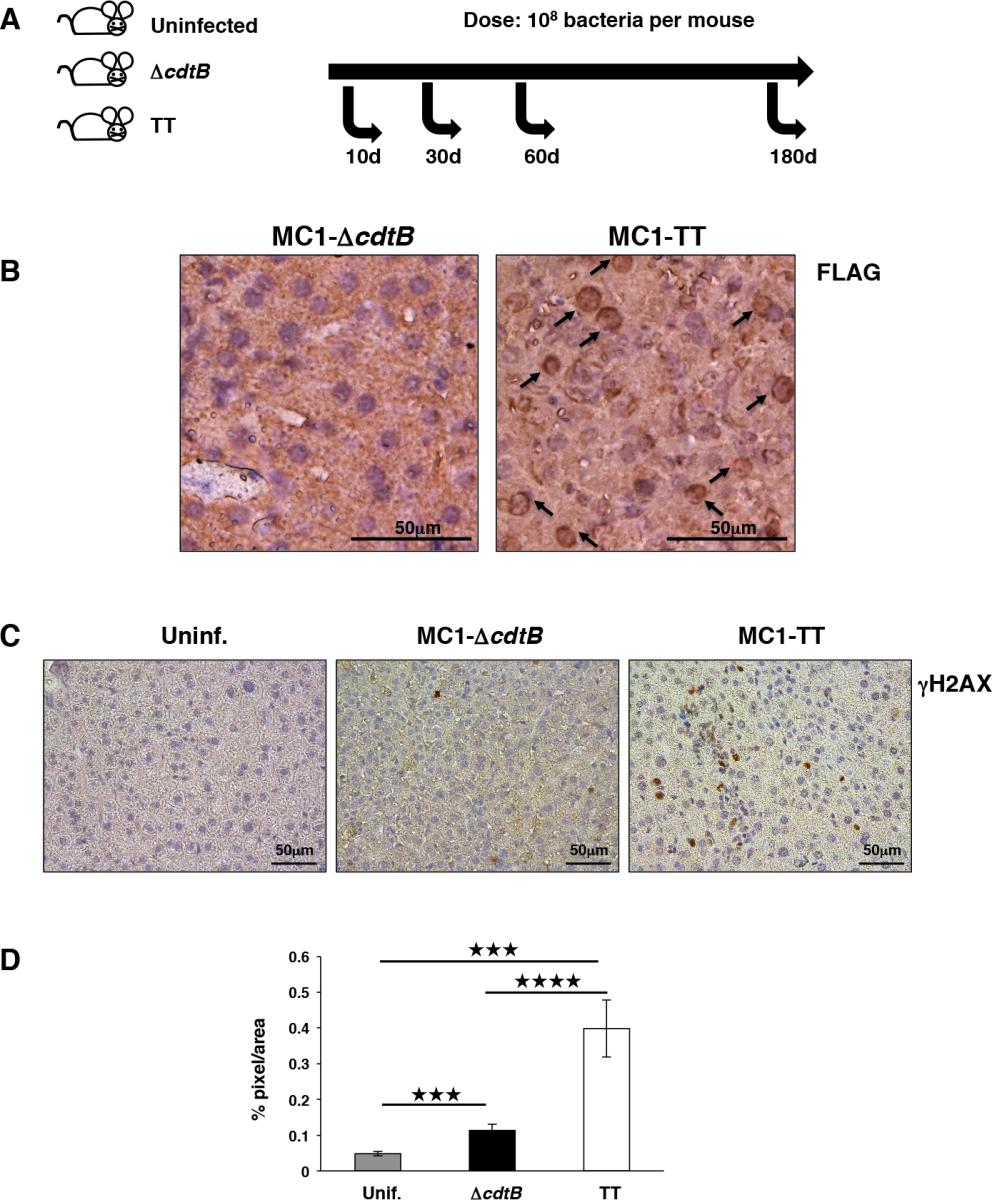
The active CdtB subunit is expressed in vivo. A. Infection model. Female 129S6/SvEvTac mice were randomized into three groups. Mice were infected orally with *S*. Typhimurium carrying the inactive (∆*cdtB*) or functional toxin operon (TT) for 10, 30, 60 and 180 days, at an infection dose of 10^8^ bacteria per mouse. Uninfected mice were gavaged with PBS. Groups of 5 to 6 mice were used, unless specified otherwise. **B.** Immunohistochemical analysis performed with an anti-FLAG-specific antibody to detect the active CdtB subunit of the typhoid toxin in liver tissue of mice infected for 10 days with the control or toxigenic MC1 strains. The black arrows indicate the nuclear localization of the toxin subunit. **C.** Immunohistochemical analysis performed with a rabbit anti-γH2AX-specific antibody to detect induction of DNA damage in liver tissue of uninfected mice or mice infected for 10 days with the control or toxigenic MC1 strains. **D.** Quantification of the γH2AX-specific staining, expressed as % of pixel per area. Statistical analysis was performed using the Student t-test, **** p value ≤ 0.0001, ***p value ≤ 0.001 (n mice = 5).

For the *in vivo* experimental set up, groups of five to six 129S6/SvEvTac mice were infected orally with 10^8^ bacteria per mouse, and euthanized 10, 30, 60 and 180 days post-infection (p.i.), as illustrated in Fig 1A. We selected this specific mouse strain, as it was previously shown to represent a suitable model to study persistent *S*. Typhimurium infections [38].

To assess the expression of the active CdtB subunit *in vivo*, we performed immunohistochemistry analysis using an anti-FLAG antibody on the liver of the mice infected with the MC1-TT strain for 10 days. Tissues from mice infected with the control MC1-Δ*cdtB* strain were used as a negative control. As shown in Fig 1B, we could detect the presence of the active subunit in the cell nucleus, where the toxin exerts its effect (Fig 1B), demonstrating for the first time the expression of the active typhoid toxin in the context of an *in vivo* infection. The expression of the CdtB subunit was further associated with increased levels of γH2AX (Fig 1C and 1D), indicating induction of DNA damage.

**Fig 2:**
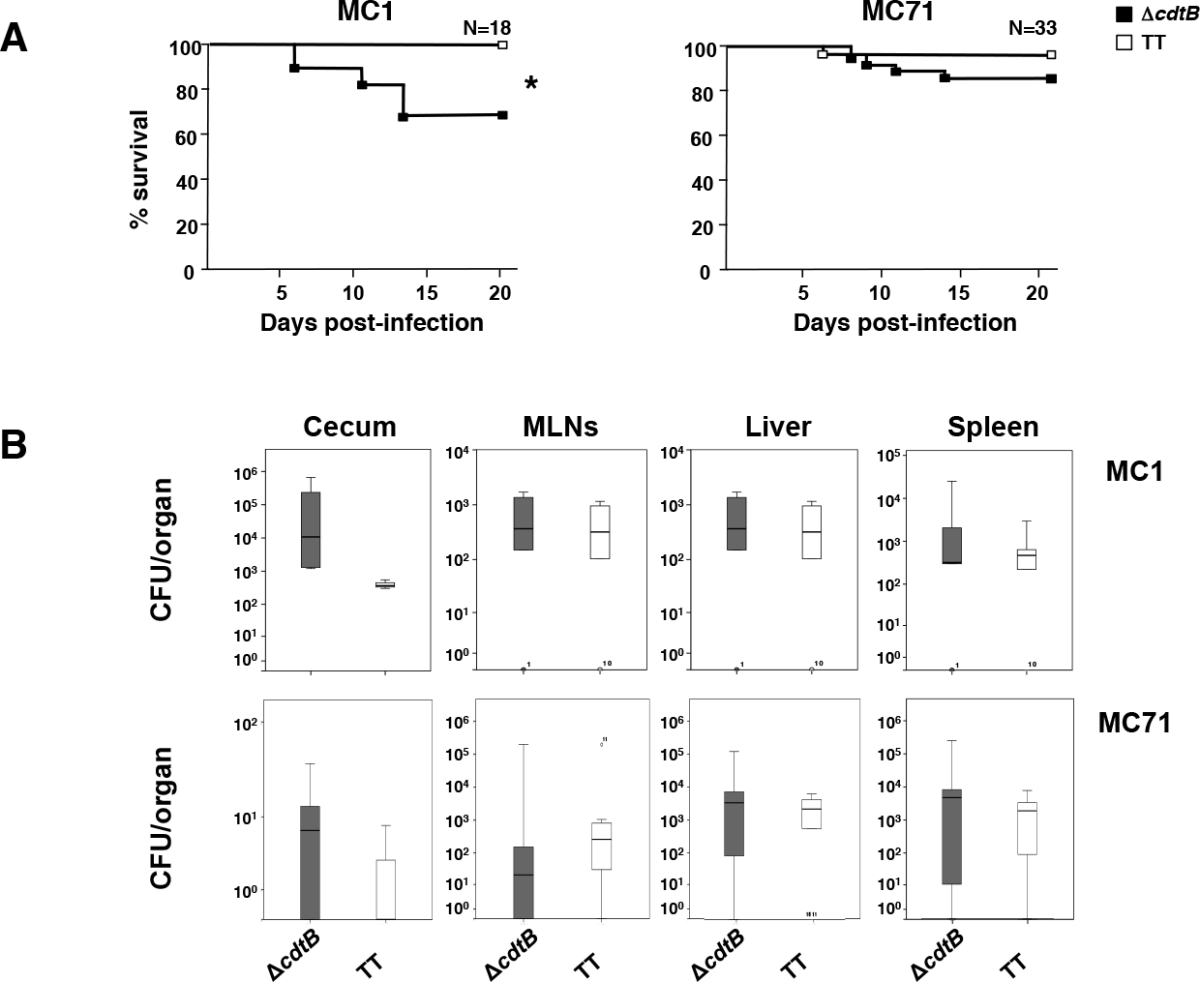
Infection with genotoxic *Salmonella* strains promotes survival of the host. **A.** Percentage of survival during the infection with the virulent MC1 (left panel) or the attenuated MC71 (right panel) strain. The Kaplan-Meier method was used to evaluate survival (95% confidence interval). *p value ≤ 0.05. **B.** Dissemination of *S*. Typhimurium in cecum, mesenteric lymph nodes (MLNs), liver, and spleen in mice infected for 10 days with the control or toxigenic MC1 or MC71 strains. Data are presented as colony forming unit (CFU) per organ. Statistical analysis was performed using the Student t-test (n mice = 6).

During the early stages of infection, approximately 40% of the mice infected with the control strain MC1-Δ*cdtB* became severely ill, similarly to previously reported data on 129S6/SvEvTac mice infected with a virulent *S*. Typhimurium strain [38], and were euthanized before the experimental end point (Fig 2A, left panel). Interestingly, the mortality rate was significantly reduced in mice infected with the MC1-TT strain (Fig 2A). A similar trend on the host survival, albeit less pronounced due to the lower mortality rate of the attenuated strain, was observed in mice infected with MC71-TT (Fig 2A, right panel).

Mice overcoming this critical period survived until the end of the experimental time course (Fig 2A), and did not present any signs of disease, independently of the infecting bacterial strain.

The reduced mortality rate in mice infected with the toxigenic strain was not associated with an impaired bacterial invasive capacity, since we did not detect statistically significant difference in bacterial recovery in the cecum, mesenteric lymph nodes, liver and spleen at 10 days p.i., independently of the presence of a functional genotoxin (Fig 2B).

### Infection with the toxigenic strain modulates the host immune response and promotes a persistent asymptomatic infection

**Fig 3:**
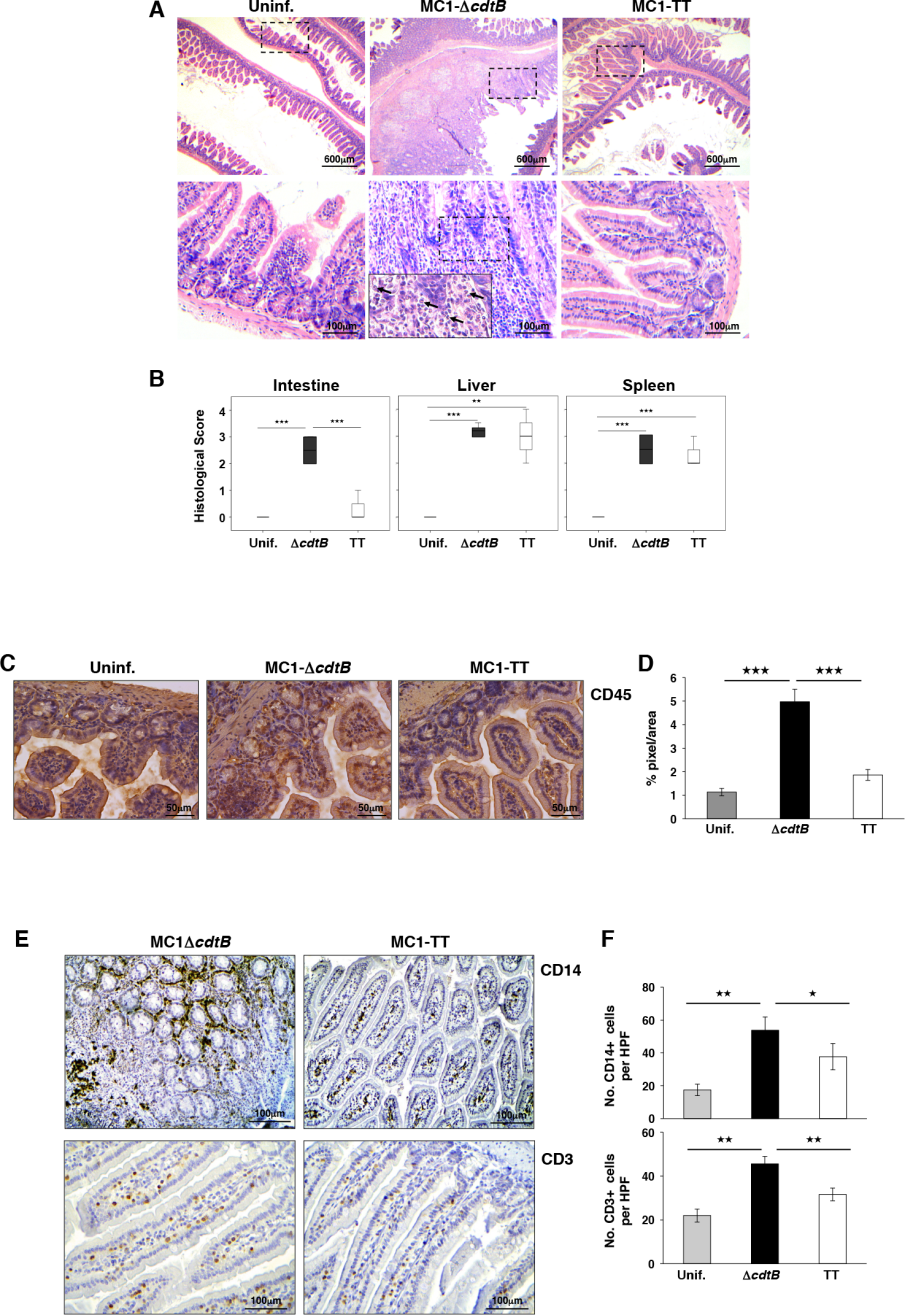
Infection with the toxigenic MC1 strain reduces the inflammatory response in the intestine. **A.** Haematoxylin and eosin staining of the intestine of uninfected mice or mice infected for 10 days with the control or toxigenic MC1 strains. Black arrows indicate accumulation of neutrophils within the lamina propria, along with invasion of the crypts. The dashed squares indicate the area that was enlarged in the middle panel. **B.** Histological scores of intestine, liver and spleen of uninfected mice, and mice infected with the control or toxigenic MC1 strains 10 days p.i. The following score was used to grade the lesions: 0: no lesions observed; 1: mild; 2: moderate; 3: severe; 4: very severe. Statistical analysis was performed using the Student t-test (n mice = 5). **C.** Immunohistochemical analysis performed with a rabbit anti-CD45-specific antibody to detect leukocyte recruitment in the intestinal tissue of uninfected mice and mice infected for 10 days with the control or toxigenic MC1 strains. **D.** Quantification of the CD45-specific staining, expressed as % of pixel per area. Statistical analysis was performed using the Student t-test (n mice = 5). **E.** Immunohistochemical analysis performed with rabbit anti-CD14- or mouse anti-CD3-specific antibodies to detect T lymphocyte and macrophage recruitment, respectively, in the intestinal tissue of mice infected for 10 days with the control or toxigenic MC1 strains. **F.** Quantification of the CD14- and CD3-positive cells (HPF: high power field). Statistical analysis was performed using the One-Way ANOVA test (n mice = 5).

**Fig 4:**
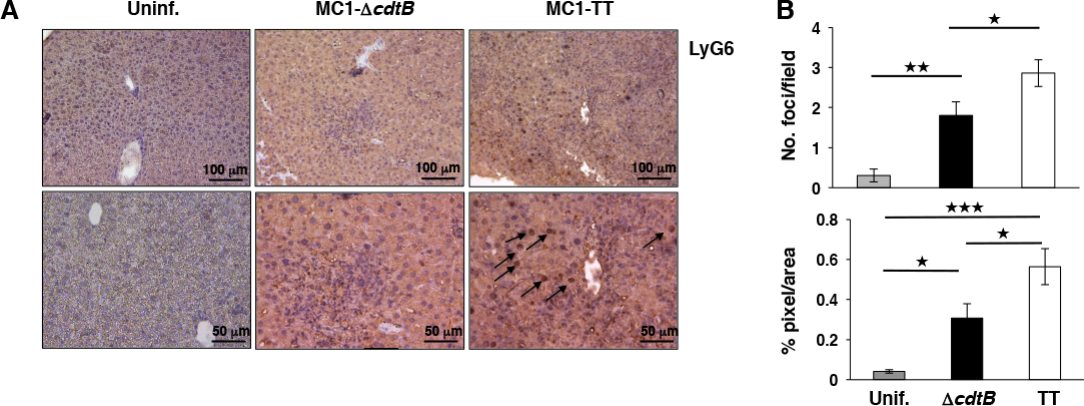
Infection with the toxigenic MC1 strain enhances the inflammatory response in the liver. **A.** Immunohistochemical analysis performed with an anti-LyG6-specific antibody to detect granulocytes and neutrophil recruitment in the liver tissue of uninfected mice and mice infected for 10 days with the control or toxigenic MC1 strains. **B.** Quantification of the number of inflammatory foci per field (left panel) and the quantification of the LyG6-specific staining (right panel), expressed as % of pixel per area. Statistical analysis was performed using the Student t-test. (n mice = 5). ***p value ≤ 0.001, **p≤ 0.01, and *p ≤ 0.05 (n mice = 5).

Since the most significant effects on the host survival were observed in mice infected with the virulent strain (MC1), we continued a more detailed analysis using this infection setting. Histological analysis demonstrated that infection with the toxigenic strains was associated with a significant decrease of severe enteritis 10 days p.i. (Fig 3A and 3B). This effect was confirmed by immunohistochemical analysis performed with antibodies specific for CD45, CD14 and CD3, which demonstrated a reduced recruitment of leukocytes (Fig 3C and 3D), macrophages and T lymphocytes (Fig 3E and 3F), respectively in the mucosa of mice infected with the MC1-TT strain compared to the levels observed in mice infected with MC1-Δ*cdtB*. Conversely, livers and spleens showed moderate to severe levels of inflammation, independently of the strain used (Fig 3B). However, a more detailed analysis demonstrated that the expression of the genotoxin in the liver was associated with an increase in the number of inflammatory foci and enhanced infiltration of myeloid cells, including granulocytes and neutrophils, as assessed by immunohistochemical analysis using anti-LyG6 specific antibody (Fig 4A and 4B). Thus, the presence of the gene encoding the CdtB subunit promotes a tissue specific modulation of the host immune response, with a clear suppressive effect in the intestine and an enhancement of the inflammatory response at systemic sites.

**Fig 5:**
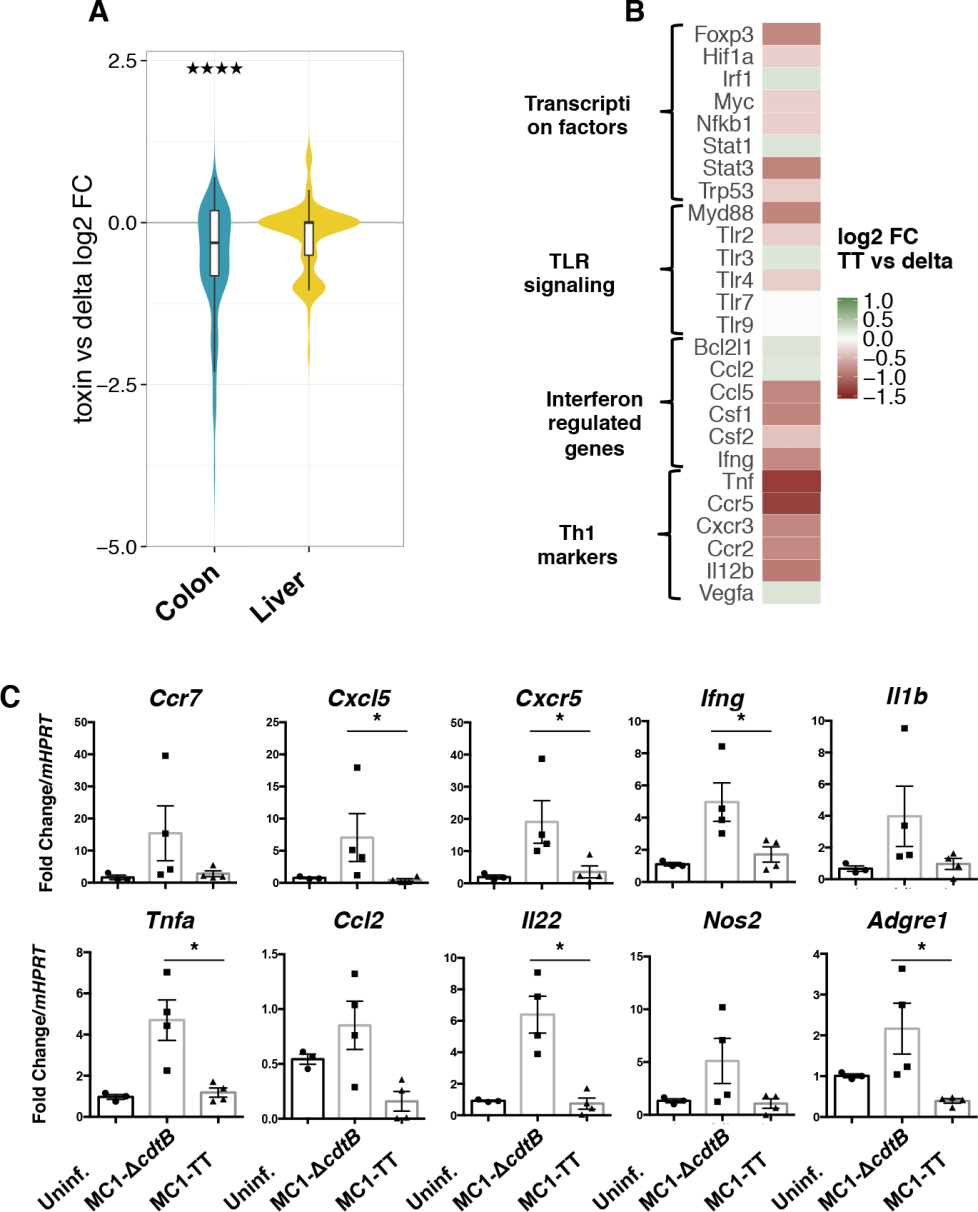
qPCR-Array analysis of colon and liver 10 days p.i. qPCR-Array analysis of 84 genes involved in inflammation and immunity in colon and liver of mice infected for 10 days with the MC1-∆*cdtB* or MC1-TT strains. **A.** Violin plot representing the distribution of the log_2_ fold changes of transcripts in the indicated tissues of mice infected with the MC1-TT strain compared to those detected in mice infected with the MC1-∆*cdtB* strain. Statistical analysis was performed with the Wilcoxon rank-sum test. Colon: p-value = 4.047e-09 (the distribution of the log2 fold changes is significantly shifted below 0). ****p value ≤ 0.0001. **B.** Heat map and functional annotation analysis for genes down- and up-regulated identified in A. **C.** Expression levels of the indicated genes in the colon of uninfected mice and mice infected with the indicated *Salmonella* strains for 10 days assessed by qPCR analysis. The data are presented as fold change relative to the housekeeping gene *Hrpt*. Statistical analysis was performed using non-parametric Mann-Whitney test. *p value ≤ 0.05 (n mice = 4).

The immunomodulatory effect of the typhoid toxin was further analyzed at the level of gene expression, using a qPCR array specific for 84 genes involved in inflammation and immunity. The data are presented in Fig 5A as the ratio of the levels of transcripts expressed in mice infected with the MC1-TT strain compared to those detected in mice infected with the control strain at 10 days p.i. Most of the genes whose expression was significantly altered by the infection with the toxigenic strain were downregulated in the colon (Fig 5A), confirming the histological data (Fig 3). Infection with the MC1-TT strain did not impose major changes in the gene expression pattern of the liver (Fig 5A).

Functional annotation analysis highlighted colon-specific downregulation of gene sets involved in the activation of transcription factors, Toll-like receptor (TLR) signaling, interferon response, and Th1-mediated responses (Fig 5B). Fig S2 shows the complete list of the genes significantly de-regulated in a toxin-dependent manner. qPCR analysis validated the toxin-induced significant suppression of a set of genes included in the array (*Cxcl5*, *Cxcr5*, *Ifng*, and *Tnfa*), and showed a similar downregulation for a panel of genes not present in the array, but strongly associated with a pro-inflammatory response (*Il22* and *Adgre1*) (Fig 5C). A similar trend, although statistically not significant was observed for other proinflammatory genes analyzed, such as *Ccr7*, *Il1b*, *Ccl2* and *Nos2* (Fig 5C). Interestingly, the decreased mRNA levels of the macrophage marker *Adgre1* were paralleled by a reduced recruitment of macrophages as detected by immunohistochemistry analysis (compare Figs 3E and 3F with Fig 5C).

We further performed a genome wide transcriptomic analysis of the tissues from mice infected with the attenuated MC71 strain at 60 days p.i. Similar to the data presented in Fig 5, we observed that the transcription of the majority of the genes was downregulated in the intestine of mice infected with the MC71-TT strain (log_2_ fold change < -1) as compared to the levels observed in mice infected with the control strain (S3A Fig). When we analyzed terms that were significantly enriched within the up-regulated transcripts, we observed terms associated with negative regulation of the host immune response in the colon (S3B Fig). These results were validated by qPCR analysis performed on selected genes involved in the activation of a pro-inflammatory response. The data showed a toxin-induced significant decrease in *Tnfa* and *Adgre1* gene expression (S4A Fig). A similar trend, although not statistically significant, was observed for the *Il1b* transcript. Interestingly, the pattern of the liver transcriptome was different, demonstrating that the expression of the CdtB subunit is involved with both upregulation and downregulation of gene transcription (S3A Fig). Gene ontology analysis of the up-regulated genes showed a significant enrichment in genes associated with activation of the host defense response (S3B Fig). This conclusion was further confirmed by detecting significantly higher mRNA levels of genes encoding proinflammatory molecules, such as IL1β (*Ιλ1b*), IL6 (*Il6*), iNOS (*Nos2*) and the macrophage marker F4/80 (*Adgre1*) in the liver of mice infected with the MC71-TT strain compared to the levels observed in mice infected with MC71-Δ*cdtB* (S4B Fig).

**Fig 6:**
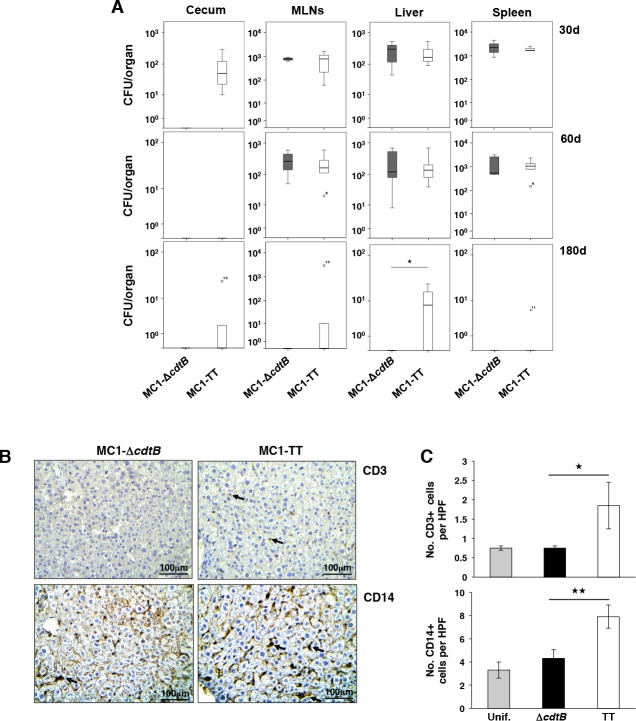
The presence of the genotoxin gene promotes the establishment of a persistent infection. **A.** Dissemination of *S*. Typhimurium in cecum, mesenteric lymph nodes (MLN), liver, and spleen in mice infected for 30, 60 and 180 with the MC1-∆*cdtB* or MC1-TT strains. Data are presented as colony forming unit (CFU) per organ. Statistical analysis was performed using the Student t-test. *p value ≤ 0.05 (n mice = 6). **B.** Immunohistochemical analysis performed with rabbit anti-CD3- or mouse CD14-specific antibodies in the liver of mice infected for 180 days with the control or toxigenic MC1 strains. Arrows indicate the CD3+ and CD14+ immune cells respectively **C.** Quantification of the CD3- and CD14-positive cells (HPF: high power field). Statistical analysis was performed using the One-Way ANOVA test, **p≤ 0.01, and *p ≤ 0.05 (n mice = 4).

**Fig 7:**
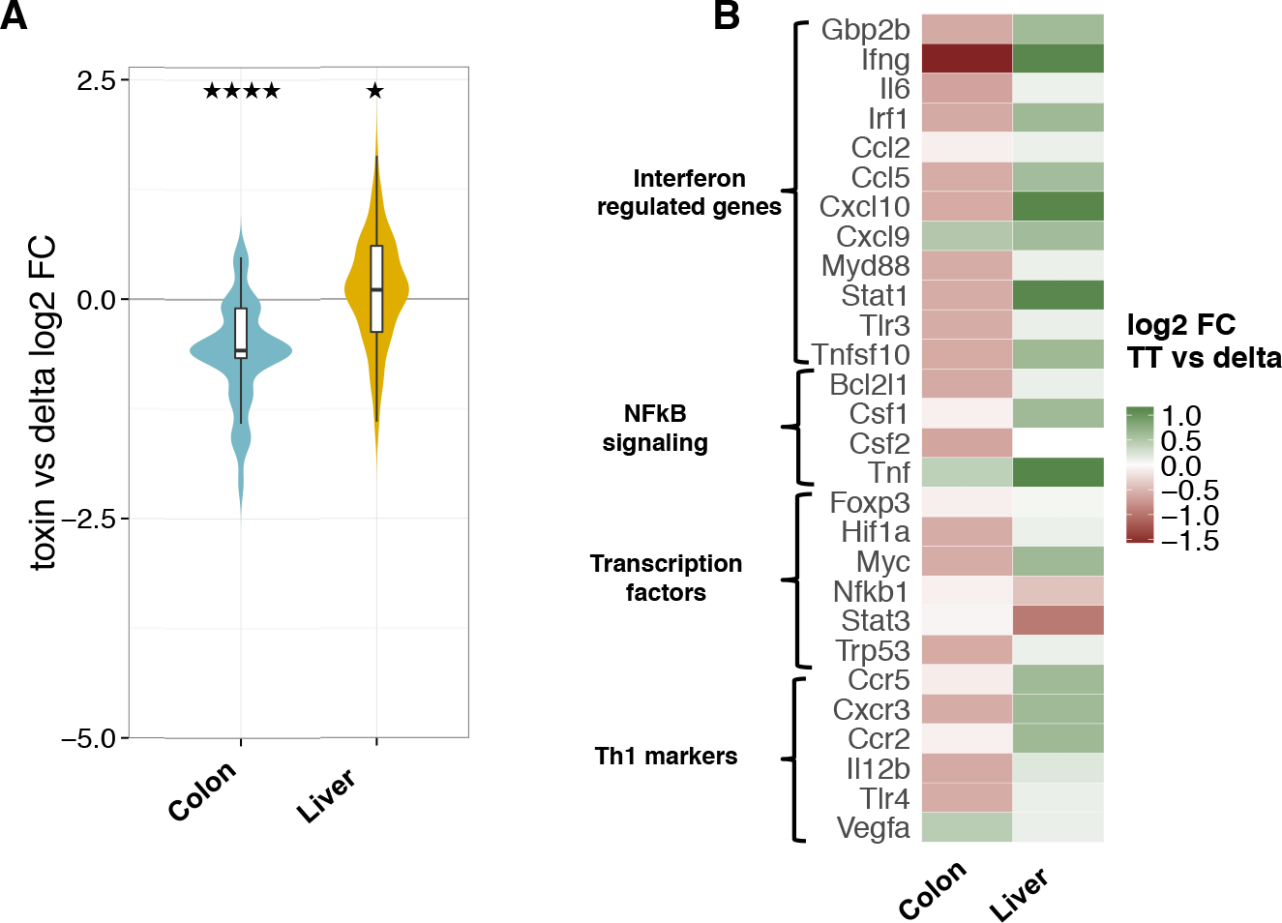
qPCR-Array analysis of colon and liver 180 days p.i. **A.** Violin plot representing the distribution of the log_2_ fold changes of transcripts in the indicated tissues of mice infected with the MC1-TT strain compared to those detected in mice infected with the MC1-∆*cdtB* strain at 180 days p.i. Statistical analysis was performed with the Wilcoxon rank-sum test. Colon: p-value = 4.989e-13 (distribution of log2 fold changes is significantly shifted below 0). Liver: p-value = 0.03676 (distribution of log2 fold changes is moderately shifted over 0). ****p value ≤ 0.0001, *p ≤ 0.05. **B.** Heat map and functional annotation analysis for genes down- and up-regulated identified in A.

We next assessed whether the presence of the genotoxic subunit has any impact on the later stages of infection. To this end, we have evaluated the bacterial burden in infected mice during a period from 30 to 180 days p.i. As shown in Fig 6A, we recovered equal amounts of bacteria at 30 and 60 days p.i., independently of the presence of an active CdtB subunit, with the exception of the cecum, where occasionally we recovered only MC1-TT bacteria, expressing the active toxin, suggesting intestinal colonization. However, at 180 days p.i., we still detected a substantial amount of bacteria in the liver of 83% of the mice (5 out of 6) infected with the toxigenic MC1 strain (Fig 6A), and sporadically, we also recovered bacteria from the cecum and mesenteric lymph nodes. In contrast, we could not detect bacteria in the organs of mice infected with the control MC1 strain at 180 days p.i. (Fig 6A), indicating that the presence of the genotoxin in this strain of *S*. Typhimurium promotes the establishment of an asymptomatic long-term infection. Histologically, the chronic carrier status was not associated with major changes in the tissue architecture, however, immunohistochemical analysis showed a significant increase in T lymphocyte (CD3) and macrophage (CD14) infiltration in the liver of mice infected with the toxigenic strain compared to the levels observed in mice infected with the control strain (Fig 6B and 6C), suggesting a higher degree of activation of the host response. In line with these results, qPCR array analysis of 84 genes regulating inflammation and immunity showed a moderate upregulation of genes associated with activation of the host immune response (Fig 7A). Functional annotation analysis highlighted liver-specific upregulation of gene sets associated with interferon and NFκB signaling, transcription factors, and Th1 responses in the liver of mice colonized with the toxigenic strain (Fig 7B). An opposite profile, characterized by a significant downregulation of gene transcription, was detected in the colon (Fig 7A and 7B), similarly to the pattern observed 10 days p.i.

Collectively, these data indicate that infection with a genotoxic *S*. Typhimurium suppresses severe enteritis and promotes the survival of the host in the early stages of infection, and favours the establishment of a long-term asymptomatic infection.

### Genotoxic *Salmonella* alters the intestinal microbiota

The intestinal microbiota has been shown to modulate the host immune system and influence the colonization of pathogenic bacteria [39–41]. Therefore, we assessed whether the suppression of the intestinal inflammatory response observed in mice infected with the toxigenic MC1 strain was associated with specific changes in the gut microbiota structure. We performed a high-throughput 16S rRNA gene sequencing of bacterial DNA extracted from stool samples, collected from uninfected mice, and mice infected with the MC1-TT or the MC1-Δ*cdtB* strain at 10, 60 and 180 days p.i. A total of 35 stool samples were analyzed, resulting in 2,014,982 high-quality sequence reads, with a mean of 57,571 reads per sample (range, 3,454–181,414). Reads were clustered into 7,614 operational taxonomic units (OTUs) at 97% identity. S5A Fig shows the rarefaction curves of the ecosystem alpha diversity, which approximated saturation for all samples analyzed, indicating that the observed number of identified taxa is close to the expected value.

**Fig 8:**
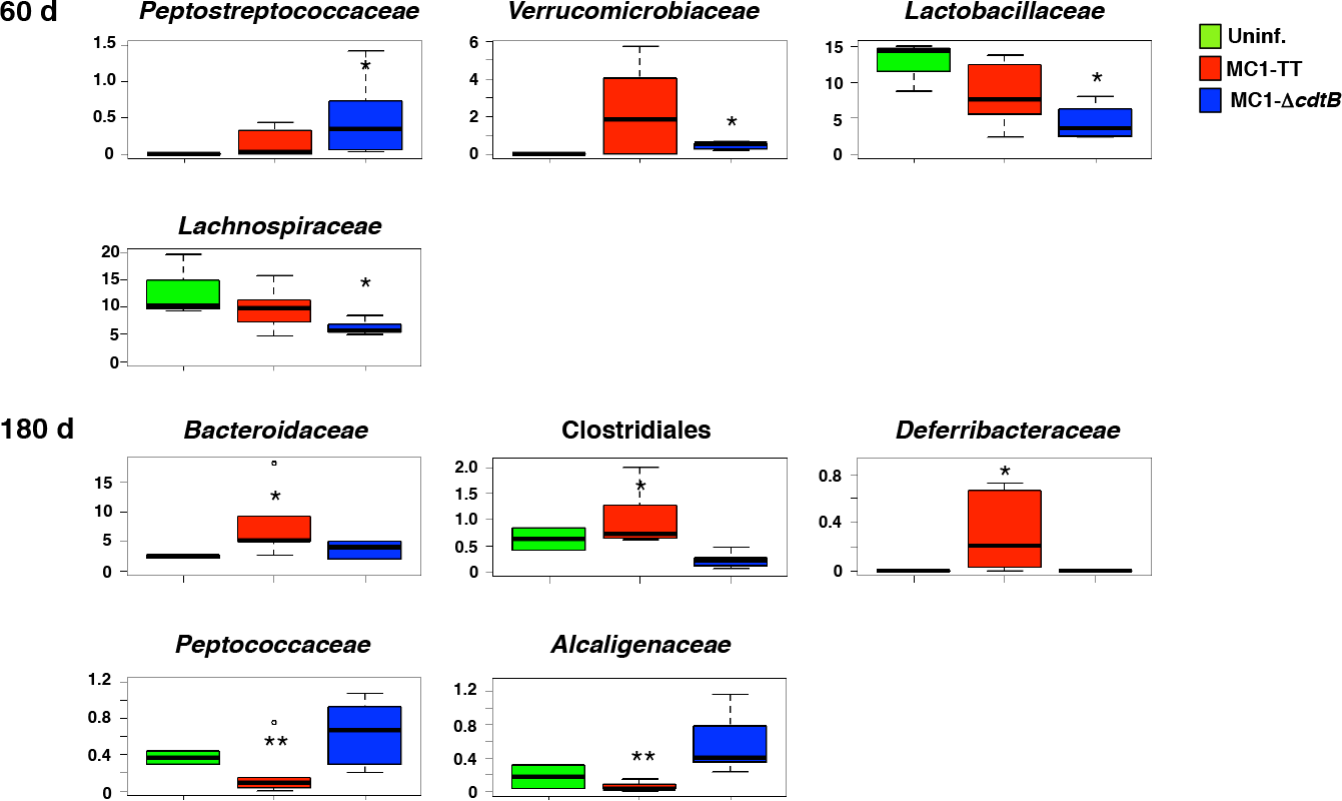
Phylogenetic analysis of the intestinal microbiota. Relative abundance of the significantly altered bacterial families in the intestinal microbiota of mice infected with the MC1-TT (TT) or MC1-Δ*cdtB* (Δ*cdtB*) strain compared to uninfected mice. *p value ≤ 0.05.

Even though infected and uninfected mice showed comparable levels of microbiome alpha and beta diversity (S5A Fig), the comparison of the compositional structure of the gut microbiota allowed us to highlight the impact of infection with the toxigenic or control strain on the mouse gut microbiome (Figs 8 and S5B). The infection with the control strain resulted in a dysbiosis with a statistically significant increase in *Peptostreptococcaeae* (p = 0.03), and depletion in *Lactobacillaceae* (p = 0.04) and *Lachnospiraceae* (p = 0.04), compared to uninfected mice or mice infected with the toxigenic strain (Fig 6) at 60 days p.i. Conversely, a statistically significant increase in *Bacteroidaceae* (p = 0.04), unclassified *Clostridiales* (p = 0.005) and *Deferribacteraceae* (p = 0.01), and lower abundance of *Peptococcaceae* (p = 0.03) and *Alcaligenaceae* (p = 0.01) were observed in the microbiota of MC1-TT infected mice compared to uninfected mice or animals infected with the control strain (Fig 8).

In conclusion, the presence of an active genotoxin is associated with a different timing and pattern of the gut microbiota configuration, characterized by the increase of mutualistic beneficial groups (*Bacteroidaceae)* and limiting the reduction of families that exert anti-inflammatory effects (*Lactobacillaceae*, *Lachnospiraceae*).

## Discussion

Genotoxins are a family of recently identified bacterial toxins, and in spite of the extensive characterization of the responses induced in the host cells *in vitro* (reviewed in [1]), very little is known about the role of these effectors in the context of *in vivo* infections. Furthermore, while several studies have been conducted in infection models with CDT-producing bacteria (reviewed in [6]), the contribution of the typhoid toxin to disease and pathogenesis is still poorly characterized.

The present work contributes to our understanding of the role of the typhoid toxin in the context of a natural infection. We developed a model where we can perform infections with toxigenic and control *S*. Typhimurium strains in mice that have a fully functional immune system and an unperturbed microbiota, since both of these components can play synergistic roles in the acute and persistent effects of the infection process (reviewed in [41,42]). We demonstrated that the active genotoxic subunit of the typhoid toxin is expressed *in vivo* (Fig 1), and its expression correlates with a reduced mortality rate in the early phase of infection, and promotes the establishment of a persistent asymptomatic infection (Figs 2 and 6). However, in our experimental conditions, chronic infection was not associated with induction of dysplasia or pre-carcinogenic lesions within the study period.

The histological and transcriptional analysis further demonstrated that infection with the toxigenic strain was associated with a negative regulation of the inflammatory response in the intestine (Figs 3, 5, S3 and S4).

### Role of the typhoid toxin in the early phases of infection

Induction of cell cycle arrest, apoptosis or senescence in acute intoxication or infection with genotoxin-producing bacteria in a broad panel of cell lines suggests that expression of genotoxins associates with enhanced symptoms [2]. However, this assumption is not consistently supported by experimental data. Indeed, CDT is not required for pustule formation by *H*. *ducreyi* in experiments conducted in human volunteers [43]. Similarly, no major differences were observed in the initial phases of infection with the toxigenic strains in mouse models of enteric infection with *C*. *jejuni* or *Helicobacter* sp., [26–29,44,45]. Even less data is available regarding the role of the typhoid toxin, in acute or persistent infections. Very recently, Song and co-workers succeeded to intraperitoneally infect immunodeficient Rag2^-/-^ γc^-/-^ mice engrafted with human fetal liver hematopoietic stem cells with *S*. Typhi [33]. The authors showed a significantly higher recovery of a non-toxigenic mutant strain in the liver and spleen of the humanized mice, suggesting that a functional genotoxin would contribute to reduced bacterial replication, possibly favouring the establishment of a persistent infection. In our model, we recover a similar number of bacteria in the different organs up to 60 days p.i., regardless of the presence of an active genotoxin (Fig 6). However, the absence of the *cdtB* gene was associated with an enhanced mortality rate of the infected mice within the first 10–15 days p.i. (Fig 2). It is noteworthy that the toxin effect on the host survival was more significant in mice infected with the fully virulent MC1 strain compared to that observed in mice infected with the isogenic attenuated mutant MC71 (Fig 2). It is possible that the protective effect of the CdtB subunit can be more prominently highlighted when the infection promotes more severe signs of the disease.

The effects observed in mice infected with the toxigenic strains are dependent exclusively on the presence of the CdtB subunit, since the control strain still maintained the second “A” subunit ADP-ribosyl transferase PltA, for which no intracellular target(s) have been identified to date.

Differently from the data reported by Song et al. [5], the presence of the CdtB subunit did not enhance the classic typhoid fever symptoms in the infected mice. It is possible that this discrepancy is due to the different mouse strains used: C57BL/6, which is highly susceptible to *S*. *enterica* infection due to a polymorphism of the *Nramp1* gene [5], versus sv129 (this study). However, we did infect a limited number of C57BL/6 mice (5 mice per group) without observing any aggravation of the symptoms upon infection with the MC71-TT strain up to 10 days p.i. On the contrary, we confirmed the pro-survival effect induced by the presence of the *cdtB* gene (S6 Fig). Differently from Song and colleagues, who administered 10 μg of a highly purified preparation of the typhoid toxin intravenously [5], we used an oral infection, which mimics the natural route of *Salmonella* infection. Since very limited data are available concerning the regulation of the toxin expression and the amounts produced during an *in vivo* infection (Fig 1), intravenous injection may represent an over dosage or may deliver this effector in a different compartment, thus explaining the discrepancy of the results. However, we cannot exclude that additional factors produced by *S*. Typhimurium contribute to induce a host response, which differs from that promoted by the purified toxin or by the toxigenic *S*. Typhi. The effects noted by Song and colleagues can also be related to the presence of human cells in their experimental system, which may induce different host responses and bacterial replication niches. Thus, this is an issue that needs to be addressed in future experiments.

### Role of the typhoid toxin in the establishment of persistent infections

The literature supports a role of the CDT genotoxin in the establishment of a persistent infection [26–29]. Our data demonstrate that a genotoxic-competent *S*. Typhimurium can also promote long-term infections (Fig 6). Interestingly, we could detect the presence of *Salmonella* in the cecum of mice infected with the toxigenic MC1 strain at 30 and 180 days p.i. Monack and co-workers have previously shown that *S*. Typhimurium can persist in the sv129 mouse model for up to 1 year, and that the main sites of persistence are the mesenteric lymph nodes [38]. In our model, we confirmed the occasional presence of the MC1-TT strain in this tissue at 180 days p.i., however the liver appears to represent the preferred site of infection in our experimental conditions (Fig 6). Liver colonization may represent the pathway used by the persistent *Salmonella* to re-cycle via the gallbladder and recolonize the small intestine, leading to fecal shedding, as previously proposed (reviewed in [46]). However, competition experiments with tagged isogenic *Salmonella* strains have recently demonstrated that the main source of fecal shedding is represented by subpopulations that colonize the cecum and colon, while systemic subpopulations (e.g. *Salmonella* present in the liver or spleen) are excluded from these sites [47]. Furthermore, increased *Salmonella* levels in the gallbladder do not lead to fecal shedding of systemic bacteria [47]. These data indicate that only certain subpopulations of bacteria can compete with the local microbiota and establish a successful colonization of the intestinal niche, leading to enhanced shedding. It is noteworthy that we detect the presence of the MC1-TT strain in the cecum even at 180 days p.i. (Fig 6). The detection of *Salmonella* in the cecum is concomitant with a toxin-specific alteration of the intestinal microbiota in the infected mice (Fig 8). Since the intestinal microbiota can limit the colonization with pathogenic bacteria [41], it is possible that the presence of the *Salmonella* MC1-TT in this niche is favoured by the specific ecosystem profile promoted by the presence of the typhoid toxin.

### Typhoid toxin as immune-modulator

Recently, it has become clear that ionizing radiation, a well-known DNA damaging agent, exerts a prominent effect in regulation of the immune response (ImR). The main purpose of the cross-talk between DDR and ImR is to ensure protection of the host from dangerous insults [48,49]. The histological and transcriptomic analysis shown in Figs 3, 4, 5, 6 and 7 revealed a complex effect of the typhoid toxin on the regulation of the host immune response in short and long-term infections. We observed a decreased inflammatory response in the colon, while there was a trend to promote an enhanced inflammatory profile in the liver of the mice infected with the toxigenic MC1 strain. It is well established that the initial stages of infection by *S*. Typhi in humans are usually asymptomatic due to a suppression of the inflammatory response (reviewed in [42]), allowing the bacterium to establish a systemic and persistent infection, while limiting the host tissue damage. Interestingly, *S*. Typhi may have developed several strategies to ensure a stealth invasion of the host through the intestinal mucosa. Several reports demonstrated that the *S*. Typhi Vi capsular antigen strongly suppresses secretion of pro-inflammatory mediators, such as IL8, *in vitro* and in *ex vivo* colonic tissue explants [50,51]. Knock-in gene expression of the Vi antigen in a model of *S*. Typhimurium inhibits recruitment of NK cells and neutrophils in spleens and mesenteric lymphnodes (MLN) of mice orally infected for 24h. This effect correlated with a decreased percentage of MIP-2, TNFα and IFNγ producing cells in spleen and MLN 24h post-infection with the Vi expressing *S*. Typhimurium compared to the effects induced by the isogenic Vi negative strain [52]. Furthermore, expression of either the *S*. Typhi specific *viaB* locus, which encodes for the regulatory gene *tviA*, biosynthesis *tviBCDE* and export genes *vexABCDE* (involved in production of the Vi antigen) or the single *tviA* gene in *S*. Typhimurium reduces the host inflammatory response in a bovine ligated ileal loop model [53].

While the Vi capsular antigen exerts its immunosuppressive effects by interacting with the membrane proteins prohibitin and prohibitin-related molecule BAP-37, thus altering the host cellular signal transduction pathways [50], the typhoid toxin may suppress the host immune response in the intestinal mucosa due to its effects on cells of the immune system. Indeed in *in vitro* experiments, CDTs block proliferation and induce apoptosis of T- and B-lymphocytes, inhibit IFN-γ secretion of T lymphocytes, induce apoptosis of monocytes and immature monocyte-derived dendritic cells (DCs) and impair the stimulatory activity of DCs, the key activators of the adaptive immune responses [13,14,54–58]. Furthermore, intravenous injection of purified typhoid toxin is associated with a dramatic decrease in the number of white blood cells [5]. A recent observation demonstrated that induction of DNA damage via different mechanisms, such as oxidative burst, ionizing radiation or etoposide, strongly inhibits the secretion of pro-inflammatory cytokines in purified human neutrophils activated by several pathogen molecular patterns (e.g. LPS, flagellin and zymogen). Thus, it is possible that the DNA damaging activity of the typhoid toxin promotes a similar effect in the intestine [59].

It is noteworthy that anti-inflammatory properties have been previously reported for another bacterial genotoxin, colibactin, encoded within the *psk* genomic island of the probiotic *E*. *coli* strain Nissle 1917. A non genotoxic mutant carrying a deletion of the *cblA* gene is impaired in its ability to prevent colitis in two models: the Dextran Sodium Sulphate (DSS)-induced acute colitis in rats and a T-cell dependent model of chronic colitis induced by the adoptive transfer of naive CD4^+^ CD45RB^high^ T cells in immunocompromised SCID mice [60].

We cannot exclude that the observed changes in the intestinal microbiota also contribute to a more pronounced tissue damaging response in the intestine of mice infected with the control strain. Indeed, we detected a pro-inflammatory configuration of the gut microbiota ecosystem, being enriched in *Peptostreptococcaeae*, and depleted for *Lactobacillaceae* and *Lachnospiraceae* in mice infected with the MC1 control strain (Fig 8) [61–63]. Conversely, mice infected with the toxigenic strain were characterized by long-term changes that favour the increase of important mutualistic components of the intestinal microbial ecosystem, such as *Bacteroidaceae* [64], as well as the maintenance of healthy-like levels of immunomodulating families such as *Lactobacillaceae* and *Lachnospiraceae*. A possible role for the microbiota in the anti-inflammatory effect of the toxigenic strain may also explain why we observed it specifically in the intestine, but not in the liver or spleen, where infection with the MC1-TT strain was eventually associated with a pro-inflammatory environment (Figs 3, 6 and 7).

We cannot exclude that this tissue specificity may be dictated by the tissue-based class control, more prone to suppress a strong pro-inflammatory and tissue-damaging Th1 response in the intestine, as proposed by Matzinger and co-authors [65]. The transcriptional analysis presented in Figs 5 and 7 is consistent with a downregulation of Th1 markers, interferon and NFκB signaling as well as inflammatory cytokines and chemokines/chemokine receptors [66–69].

In conclusion, our data shed some light on the effects of the typhoid toxin, a still poorly characterized microbial effector, in the complex interplay between the host and the pathogenic bacteria, and further pose a semantic and biological question on whether this effector can be defined as “toxin”.

## Materials and Methods

### Bacterial strains

The *S*. Typhimurium strains MC1 and MC71 were previously described [35]. The genes encoding the typhoid toxin (TT) or a toxin carrying a deletion of the *cdtB* subunit (Δ*cdtB*) were cloned into the pEGFP-C1 plasmid (Clontech Laboratories, Mountain View, CA, USA) as described in [70]. The gene encoding the chloramphenicol resistance (Cm) was amplified from the pKD3 plasmid (NCBI Gene Bank AY048742), using the following primers: 5’-AAAGGATCCGTGTAGGCTGGAGCTGCTTC-3’ and 5’-AAAGGTACCCATATGAATATCCTCCTTAG-3’, and cloned into the *BamHI-KpnI* sites of the pEGFP-C1-TT or pEGFP-C1-Δ*cdtB* plasmids. The toxin and the chloramphenicol genes were amplified using the primers 5’-GTCCGCACGTTCTTCCGTGGCGTGGATATTAGTCAGGTCTTTAGCGCCAAAGATATTGCCATTCTGTAACTGATAAAGTAGGTGTGCTTA-3’ and 5’-AAT GCCGCTTTTAATGAGTCGATGGACACGACGCCCACGAATTTATTGCATATGAATATCCTCCTTAG-3’, and transferred by homologous recombination into the genomic *proV* gene of *S*. Typhimurium strain LB5010 [71] carrying the pKD46 plasmid [72]. The MC1/MC71-TT and MC1/MC71-Δ*cdtB* strains were produced by P22int phage transduction, and grown overnight in Luria agar plates supplemented with Cm (10 μg/ml) [73]. The construction of these strains was approved by the Swedish Work Environment Authority.

The list of strains used in this work is summarized in Table 1.

**Table 1:** 

Strain name	Resistance gene	Reference
MC1	None	[35]
MC71	None	[35]
MC1-TT	Cm^a^	This study
MC1-Δ*cdtB*	Cm	This study
MC71-TT	Cm	This study
MC71-Δ*cdtB*	Cm	This study
LB5010 pKD46-TT	Amp^b^, Cm	This study
LB5010 pKD46-Δ*cdtB*	Amp, Cm	This study

a: chloramphenicol

b: ampicillin

### Infection

Female 129S6/SvEvTac mice 6- to 8-weeks-old, kept in a pathogen-free facility, were obtained from Taconic Biosciences Inc. (Bomholt, Denmark).

The *S*. Typhimurium strains were cultured overnight in complete LB medium supplemented with Cm (50 μg/ml), harvested and resuspended in cold phosphate-buffered saline (PBS) at the desired concentration. The bacterial load was confirmed by CFU counting on LB agar plates supplemented with Cm. Five to six mice per group were gavaged with 100 μl PBS containing 10^8^ bacteria or vehicle alone. Animals were monitored twice a day for the first two weeks post-infection, and once every two days thereafter. Weight was recorded every day for the first two weeks.

Mice that reached the humane endpoint were counted as dead, and sacrificed. Intestine, liver, spleen, and mesenteric lymph nodes were collected for each mouse. The cecum, two centimetres of the proximal portion of jejunum, ileum, and colon, one third of the spleen, and approximately10 mm^3^ of each liver lobe were stored in RNALater (Sigma-Aldrich, St. Louis, MO, USA) at -80°C for RNA extraction. The remaining part of jejunum, ileum, and colon, one third of the spleen, and the central part of the liver, including the gallbladder, were fixed in 4% paraformaldehyde for histological and immunohistochemical analysis. Lymph nodes, cecum content, and the remaining parts of liver and spleen were homogenized in PBS to assess bacterial recovery on LB plates supplemented with Cm (50 μg/ml).

Single blind histological examination of liver, spleen, and intestine was commissioned to the National Veterinary Institute (Uppsala, Sweden). Statistical analysis was performed using the SPSS software with a two-tailed unpaired t-test.

### Ethical consideration

All animals were handled in strict accordance with good animal practice as defined by the relevant national animal welfare bodies, following proceedings described in EU legislation. This study was approved by the Regional Animal Studies Ethical Committee, Stockholm North, Sweden (reference number N133/13).

### Survival analysis

Statistic analysis and plotting were performed with Prism(r) software (http://www.graphpad.com/scientific-software/prism/). The log-rank test on Kaplan-Meier survival graphs was used to evaluate survival data. No censured data were generated. p-values ≤ 0.05 were considered statistically significant.

### Immunohistochemistry

Formalin-fixed paraffin-embedded 4 μm tissue sections were deparaffinised in xylene and rehydrated through a graded series of alcohol. The endogenous peroxidase activity was blocked by incubating the sections 30 min with 3% H_2_O_2_ in distilled H_2_O.

#### CD45 staining

The antigen retrieval was performed by heating the sections in citrate buffer (pH6) (10 mM citric acid, 0.05% Tween 20, pH 6.0) in a microwave oven (650 W) for 30 min. The sections were blocked in 10% FCS and 1% bovine serum albumin (BSA) in Tris-buffered saline (TBS) for 2 h at 37°C. The rabbit anti-CD45 primary antibody (ab10558, AbCam, Cambridge, UK) was incubated overnight at 4°C, at a dilution of 1:200 in 1% BSA in TBS.

#### Ly6G staining

The tissue sections were heated in citrate buffer in a microwave oven (650 W) for 20 min, and blocked in 5% FCS and 1% BSA in PBS for 2 h at RT. The rat anti-Ly6G primary antibody (127602, BioLegend) was incubated overnight at 4°C, at a dilution of 1:200 in 1% BSA-TBS.

#### H2AX staining

The tissue sections were heated in sodium citrate buffer in a microwave oven (650 W) for 15 min, and blocked in 5% FCS and 1% BSA in PBS overnight at +4°C. The rabbit anti-pH2AX primary antibody (20E3, Cell Signaling) was incubated overnight at 4°C, at a dilution of 1:500 in 1% BSA-TBS.

#### FLAG staining

The tissue sections were heated in citrate buffer in a microwave oven (650 W) for 20 min, and blocked in 10% FCS, 0.3% TritonX-100 in TBS for 1 h at RT. The rabbit anti-FLAG primary antibody (F7425, Sigma) was incubated overnight at 4°C, at a dilution of 1:100 in blocking solution.

After incubation with the primary antibodies, the slides were washed 3 times with TBS, and incubated with the HRP-conjugated goat anti-rabbit (ab6721, AbCam) or anti-rat (ab6734, AbCam) secondary antibody for 1 h at a dilution of 1:200 in TBS, supplemented with 1% BSA. Immunocomplexes were visualized with the 3,3’-diaminobenzidine (DAB) substrate kit (SK-4100, Vector Laboratories Inc., Burlingame, CA, USA), according to the instructions of the manufacturer. Sections were counterstained with hematoxylin solution (Sigma-Aldrich) and mounted with Mowiol.

Images were acquired with a Leica DM5500B microscope and Leica DFC450 camera (Leica Microsystems, Wetzlar, Germany).

Micrographs taken at 10X magnification were deconvoluted and quantification of the staining was performed with ImageJ software (using the IHC Profiler plugin). The intensity of the staining is calculated as % of pixel per area.

#### CD3 and CD14 staining

Unmasking of the antigen retrieval was performed by heat-mediated antigen retrieval method in 10mM citric acid (pH6.0). The rabbit anti-CD3 (ab5690, AbCam) and mouse anti-CD14 (ab182032, AbCam) primary antibodies were incubated overnight at 4°C, at a dilution of 1:200 and 1:1000, respectively. The indirect streptavidin-biotin-hyperoxidase method was employed for the CD3 staining. Slides were incubated with the secondary biotin-conjugated antibody at a 1:200 dilution for 30 min, followed by a 30 min incubation with the Streptavidin HRP Conjugate (18–152, Millipore; 1:200). For color development DAB (Sigma) was used and hematoxylin was employed as counterstain. For the CD14 staining the UltraVision LP Detection System was used (#TL-060-HD, Thermo Scientific, Bioanalytica, Greece) according to the manufacturer’s instructions. Evaluation of CD3 and CD14 staining was performed by counting the number of the corresponding positive immune cells per high power field (HPF, magnification 400x) as previously described [74]. Kupffer cells and sinusoidal lining cells in mouse liver served as positive control for CD14. Three independent observers carried out slide examination, with minimal inter-observer variability.

### GeneChip assay

Total RNA was extracted using the RNeasy Mini Kit (Qiagen, Hilden, Germany) according to manufacturer’s instructions. Briefly, tissue samples were homogenized in RLT Buffer containing 10 μl β-mercaptoethanol and incubated with DNase I 27 Kunitz units (RNase-Free DNase Set, Qiagen). Tissue lysates were purified on spin columns and RNA was eluted in 20 μl of distilled H_2_O. RNA quantity was estimated using the NanoDrop 2000 (Thermo Scientific, Waltham, USA). Equal amounts of total RNA extracted from the tissue of individual mice from each group were pooled.

Before analysis, the quantity and integrity of the pooled RNA samples were determined using the Agilent 2100 Bioanalyzer (Agilent Technologies, Santa Clara, CA, USA). The RNA samples were subsequently amplified using the Ovation Pico WTA v.2 RNA Amplification System (NuGEN, San Carlos, CA, USA) and biotin labelled with the Encore Biotin Module (NuGEN) in accordance with the manufacturer’s instructions. The amplified and labelled samples were hybridized to the Affymetrix GeneChip Mouse Gene 2.0 ST array (Affymetrix, Santa Clara, CA, USA). The arrays were washed and stained with phycoerythrin-conjugated streptavidin using the Affymetrix Fluidics Station 450 and the arrays scanned in the Affymetrix GeneArray 3000 7G scanner.

The CEL files resulting from the GeneChip analysis were analyzed with the R software environment for statistical computing (http://www.r-project.org/) and the Bioconductor library of biostatistical packages (http://www.bioconductor.org/). Raw data were preprocessed and normalized with the RMA method using the “oligo” package. Probe sets were mapped to transcripts using the Affymetrix mogene20 annotation data contained in the “mogene20sttranscriptcluster.db” package. Differentially expressed genes (DEGs) were determined adopting a threshold based on the log2 fold change (>1 for up-regulated, < -1 for down-regulated). The ToppGene resource [75] was used for enrichment analysis of DEGs lists, using annotations from Gene Ontology (http://geneontology.org), KEGG (http://www.genome.jp/kegg/), PFAM (http://pfam.sanger.ac.uk) databases. The significance of overrepresentation was determined using a p-value threshold of 0.05.

### RT2 profiler PCR array

Total RNA from individual mice was isolated using RNeasy Mini Kit (Qiagen), pooled in equimolar amounts for each experimental group, retrotranscribed using RT^2^ First Strand Kit (Qiagen) according to the manufacturer’s instructions and loaded onto qPCR plates (Mouse Cancer Inflammation & Immunity Crosstalk PCR Array, Cat. # PAMM-181Z, Qiagen). Cycling was performed using the following conditions: 95^°^C for 10 minutes; 95^°^C for 15 seconds and 60^°^C for 1 minute for 40 cycles. Raw Ct values were normalized to the geometric mean of five housekeeping genes (Actb, B2m, Gapdh, Gusb, Hsp90ab1). Fold changes in gene expression were calculated with the delta Ct method [76]. P-values for single genes were obtained using the t-test. P-values for shifts in expression distributions for populations of genes were obtained using the Wilcoxon rank-sum test.

### qPCR analysis

RNA was retrotranscribed using High Capacity cDNA Reverse Transcription (Life Technologies, Carlsbad, CA, USA) and RiboLock RNase Inhibitor (Life Technologies) kits according to manufacturer’s instructions. cDNA was amplified using the SYBR Green Real-Time PCR Master Mixes (Life Technology) with the following primers:

*Adgre1*:

5’-AAGGGAAGGCTTTCTTCATTG-3’; 5’-CCCCATCTGTACATCCCACT-3’;

*Tnfa*:

5’-GCCTCTTCTCATTCCTGCTTGT-3’; 5’-GGCCATTTGGGAACTTCTCAT-3’;

*Il1b*:

5’-ACAAGCTGTGCATCTTCGAC; 5’-CGGCTCCTCTGAATGAAATC-3’;

*IL6*:

5’-TCCAGTTGCCTTCTTGGGAC-3’; 5’-GTGTAATTAAGCCTCCGACTTG-3’;

*Nos2*:

5’-TGCCCCTTCAATGGTTGGTA-3’; 5’-ACTGGAGGGACCAGCCAAAT-3’;

*Cxcl5*:

5’-GCATTTCTGTTGCTGTTCACGCTG-3’; 5’-CCTCCTTCTGGTTTTTCAGTTTAGC-3’

*Cxcr5*:

5’-ACTCCTTACCACAGTGCACCTT-3’; 5’-GGAAACGGGAGGTGAACCA-3’;

*Ccr7*:

5’-GAGGAAAAGGATGTCTGCCACG-3’; 5’-GGCTCTCCTTGTCATTTTCCAG-3’;

*Hprt*:

5’-GCCCTTGACTATAATGAGTACTTCAGG-3’; 5’-TTCAACTTGCGCTCATCTTAGG-3’.

Cycling was performed using the following conditions: 95°C for 15 sec, 95°C for 15 sec, 55°C for 30 sec, 72°C for 30 sec for 40 cycles. Three representative reverse transcriptase-negative samples were included in each plate to exclude genomic contamination. Gene expression was normalized to the *Hprt* housekeeping gene. Fold change in gene expression was calculated with the comparative Ct method [77]. p-value was evaluated using non-parametric Mann-Whitney test.

### Intestinal microbiota analysis

Total microbial DNA was extracted from fecal samples using the DNeasy Blood & Tissue kit (Qiagen) with a modified protocol, incorporating a lysis step in 2.5 ml of lysis buffer (500 mM NaCl, 50 mM Tris-HCl pH 8.0, 50 mM EDTA, 4% SDS). Samples were incubated at 100°C for 15 min prior to column purification. DNA concentration and quality were evaluated using the NanoDrop 2000 (Thermo Scientific).

The V3-V4 region of the 16S rRNA gene was amplified and the resulting amplicons were cleaned up and sequenced on Illumina MiSeq platform (Illumina, San Diego, CA, USA) using a 2×300 bp paired end protocol, according to the manufacturer’s instructions. Briefly, PCR reactions were carried out in a 25-µl final volume containing 12.5 ng of microbial DNA, 2X KAPA HiFi HotStart ReadyMix (KAPA Biosystems, Resnova, Rome, Italy), and 200 nM of S-D-Bact-0341-b-S-17/S-D-Bact-0785-a-A-21 primers [78] carrying Illumina overhang adapter sequences. Thermal cycle was as follows: initial denaturation at 95°C for 3 min, 25 cycles of denaturation at 95°C for 30 sec, annealing at 55°C for 30 sec, and extension at 72°C for 30 sec, and a final extension step at 72°C for 5 min. Amplicons were purified with a magnetic bead-based clean-up system (Agencourt AMPure XP; Beckman Coulter, Brea, CA, USA). Indexed libraries were prepared by limited-cycle PCR using Nextera technology and further cleaned up with AMPure XP magnetic beads (Beckman Coulter). Final libraries were pooled at equimolar concentrations, denatured and diluted to 6 pM before loading onto the MiSeq flow cell.

Raw sequences were processed using a pipeline combining PANDAseq [79] and QIIME [80]. After length and quality filtering with default parameters, reads were binned into OTUs at 97% identity using UCLUST [81]. Taxonomy was assigned using the RDP classifier against Greengenes database (May 2013 release). To filter out PCR errors and chimeras, all singleton OTUs were discarded. Alpha rarefaction was performed using the Faith’s phylogenetic diversity, Chao1, observed species, and Shannon index metrics. Beta diversity was estimated by computing weighted and unweighted UniFrac distances, which were used as input for principal coordinates analysis (PCoA).

All statistical analyses were performed in R. Significant differences were assessed by Wilcoxon signed rank test. A p value ≤ 0.05 was considered as statistically significant.

## Supporting Information

S1 Fig Construction of the *S*. Typhimurium strains MC1 and MC71 expressing the typhoid toxin. **A.** Schematic representation of the cassette encoding for the typhoid toxin *pltB*-*pltA* and *cdtB* genes from *S*. Typhi and the chloramphenicol resistance gene (Cm). The cassette was transferred by homologous recombination into the genomic *proV* gene of the *S*. Typhimurium strains MC1 and MC71. Each typhoid toxin subunit was engineered with a unique C-terminal epitope-tag to allow detection of the protein in western blots. Control strains carry a cassette where the *cdtB* gene is deleted (Δ*cdtB)*. **B.** CaCo-2 cells were infected with the MC1-Δ*cdtB* or MC1-TT strains at the MOI 50:1. The data are presented as % of the inoculum recovered at 2h (invasion) and 24h (replication) post-infection. The incoculum is defined as the number of CFU/ml present in the bacterial suspension used for infection. Mean ± SD of three independent experiments. **C.** The recombinant MC1/MC71-Δ*cdtB* and MC1/71-TT strains were grown in LB or MM5.8 medium for 24h. The latter mimics the growth conditions of *Salmonella* containing vacuole, which are required for the toxin expression [3,70]. Expression of the typhoid toxin subunits tagged with the indicated epitopes was assessed in total cell lysates by western-blot. DnaK was used as loading control. **D.** CaCo-2 cells were infected with the MC1/71-Δ*cdtB* or MC1/71-TT strains at MOI 50:1 for 24 h. Induction of DNA damage was assessed by immunofluorescence using a mouse anti-γH2AX antibody, followed by a donkey anti-mouse secondary antibody conjugated to Alexa-488 (green). Nuclei were counterstained with DAPI (blue).
(TIF)

S2 Fig qPCR array analysis colon 10 days p.i. Identity of the de-regulated genes in the colon of mice infected with the toxigenic MC1-TT strain compared to the levels observed in mice infected with the control MC1-Δ*cdtB* strain. The value between brackets indicates the % of de-regulated genes.
(TIF)

S3 Fig Infection with the toxigenic MC71 strain alters the host response. Trascriptomic analysis was performed on jejunum, ileum, colon and liver of uninfected mice or mice infected for 60 days with the MC71-TT or MC71-∆*cdtB* strains. **A.** Log_2_ fold changes of transcripts in the indicated tissues of mice infected with the MC71-TT strain compared to those detected in mice infected with the MC71-∆*cdtB* strain. **B** Gene ontology enrichment analysis of the up-regulated transcripts identified in panel A. Blue range colors indicate significantly enriched terms.
(TIF)

S4 Fig qPCR analysis of pro-inflammatory markers. Comparison of the mRNA levels for the indicated genes in the colon (**A**) and liver (**B**) of uninfected mice, and mice infected with the indicated *Salmonella* strains for 60 days, assessed by qPCR analysis. The data are presented as fold change relative to the housekeeping gene *Hrpt*. Statistical analysis was performed using non-parametric Mann-Whitney test. ***p value ≤ 0.001, **p≤ 0.01, and *p ≤ 0.05.
(TIF)

S5 Fig Diversity of the intestinal microbiota. **A.** Alpha diversity rarefaction plots. Diversity was estimated by calculating the Chao1 measure of microbial richness, the observed number of OTUs, the Faith’s phylogenetic diversity index (PD whole tree), and the Shannon diversity index. **B.** Weighted and unweighted UniFrac Principal Component Analysis (PCoA). The two components explain 57.5 and 17.7% of the variance, respectively. Alpha and beta diversity of the intestinal microbiota were determined for uninfected mice and mice infected with the MC1-TT (TT) or MC1-Δ*cdtB* (Δ*cdtB*) strain at 10, 60 and 180 days post-infection.
(TIF)

S6 Fig Survival curve of infected C57BL/6 mice. Five female C57BL/6 mice were infected orally with the *S*. Typhimurium MC71 strain carrying the inactive (∆*cdtB*) or functional toxin operon (TT) at an infection dose of 10^4^ bacteria per mouse for 10 days. The Kaplan-Meier method was used to evaluate survival (95% confidence interval).
(TIF)
