# Supplementary figures and images for: The Typhoid Toxin Promotes Host Survival and the Establishment of a Persistent Asymptomatic Infection
Source: PLoS Pathog. 2016 Apr 7;12(4):e1005528. doi: 10.1371/journal.ppat.1005528 (PMC4824513; doi:10.1371/journal.ppat.1005528)

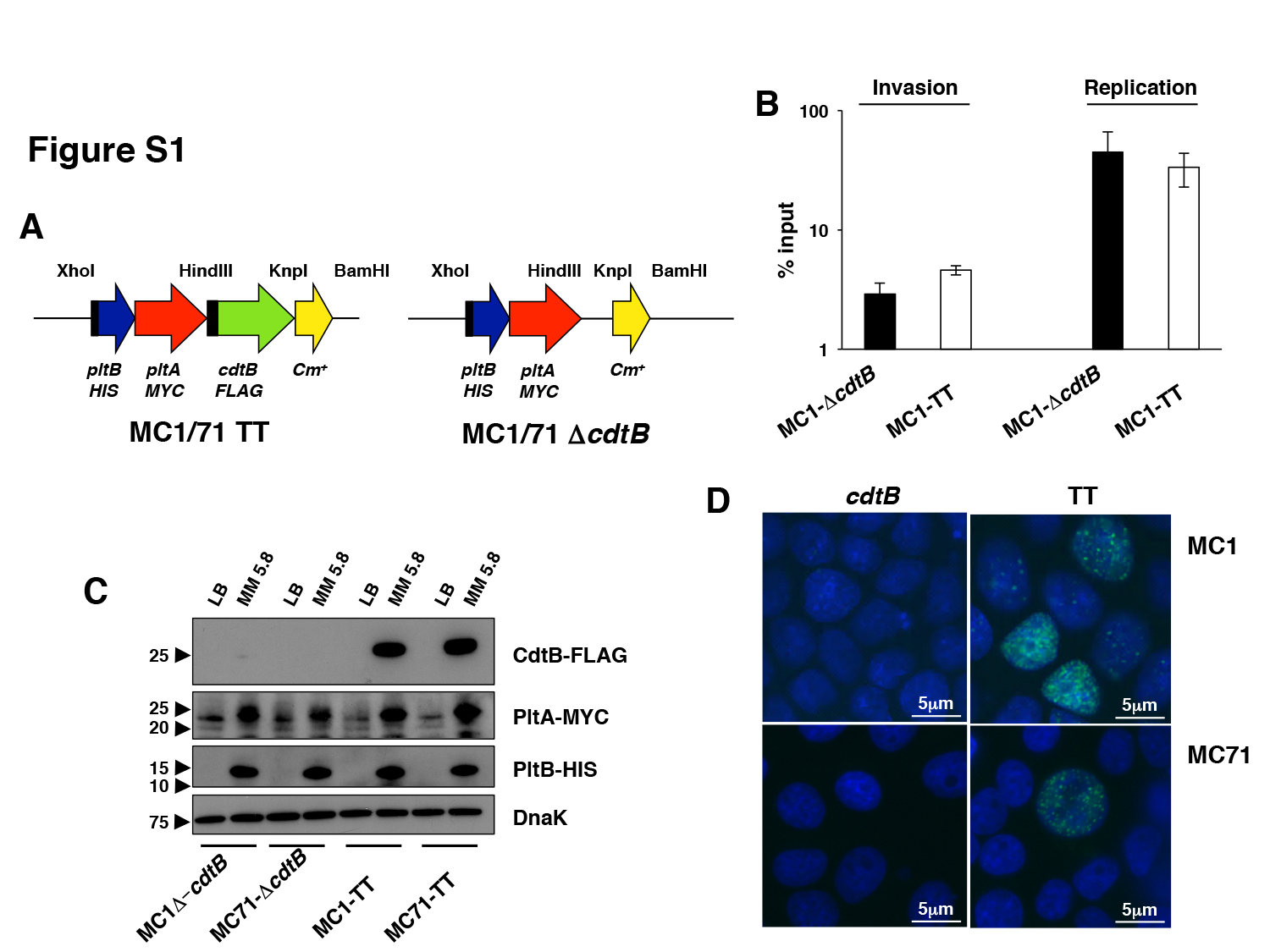

Supplement: S1 Fig — A. Schematic representation of the cassette encoding for the typhoid toxin pltB-pltA and cdtB genes from S. Typhi and the chloramphenicol resistance gene (Cm). The cassette was transferred by homologous recombination into the genomic proV gene of the S. Typhimurium strains MC1 and MC71. Each typhoid toxin subunit was engineered with a unique C-terminal epitope-tag to allow detection of the protein in western blots. Control strains carry a cassette where the cdtB gene is deleted (ΔcdtB). B. CaCo-2 cells were infected with the MC1-ΔcdtB or MC1-TT strains at the MOI 50:1. The data are presented as % of the inoculum recovered at 2h (invasion) and 24h (replication) post-infection. The incoculum is defined as the number of CFU/ml present in the bacterial suspension used for infection. Mean ± SD of three independent experiments. C. The recombinant MC1/MC71-ΔcdtB and MC1/71-TT strains were grown in LB or MM5.8 medium for 24h. The latter mimics the growth conditions of Salmonella containing vacuole, which are required for the toxin expression [3,70]. Expression of the typhoid toxin subunits tagged with the indicated epitopes was assessed in total cell lysates by western-blot. DnaK was used as loading control. D. CaCo-2 cells were infected with the MC1/71-ΔcdtB or MC1/71-TT strains at MOI 50:1 for 24 h. Induction of DNA damage was assessed by immunofluorescence using a mouse anti-γH2AX antibody, followed by a donkey anti-mouse secondary antibody conjugated to Alexa-488 (green). Nuclei were counterstained with DAPI (blue). (TIF) [file ppat.1005528.s001.tif]

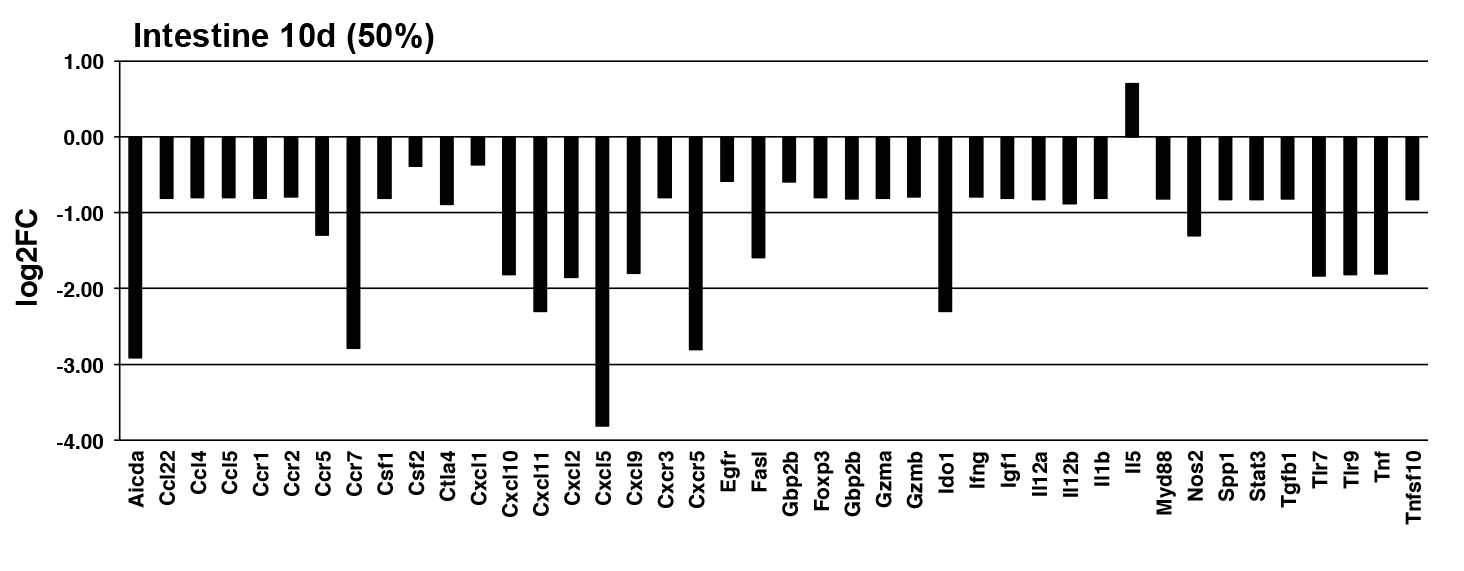

Supplement: S2 Fig — Identity of the de-regulated genes in the colon of mice infected with the toxigenic MC1-TT strain compared to the levels observed in mice infected with the control MC1-ΔcdtB strain. The value between brackets indicates the % of de-regulated genes. (TIF) [file ppat.1005528.s002.tif]

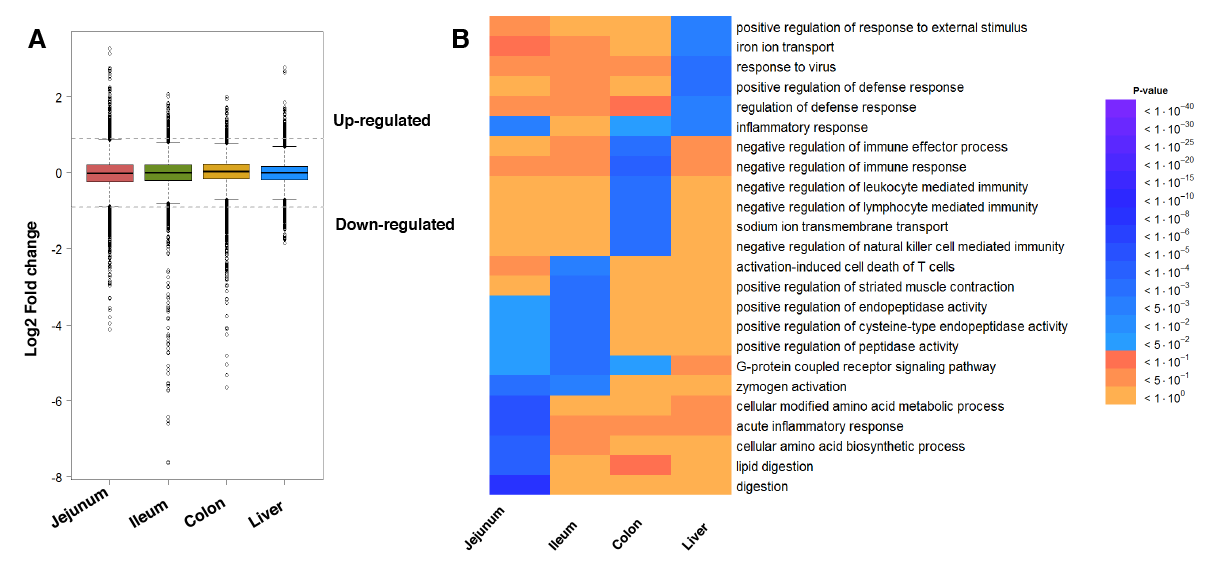

Supplement: S3 Fig — Trascriptomic analysis was performed on jejunum, ileum, colon and liver of uninfected mice or mice infected for 60 days with the MC71-TT or MC71-∆cdtB strains. A. Log2 fold changes of transcripts in the indicated tissues of mice infected with the MC71-TT strain compared to those detected in mice infected with the MC71-∆cdtB strain. B Gene ontology enrichment analysis of the up-regulated transcripts identified in panel A. Blue range colors indicate significantly enriched terms. (TIF) [file ppat.1005528.s003.tif]

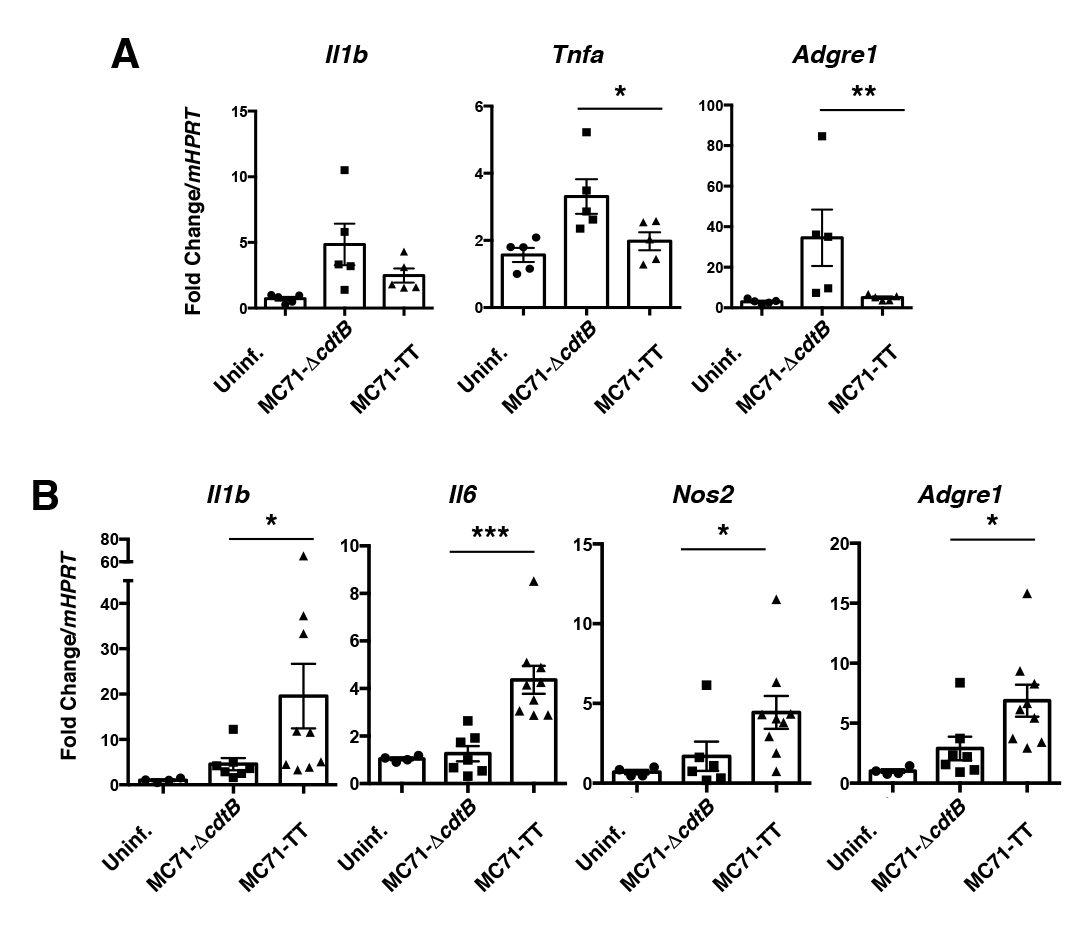

Supplement: S4 Fig — Comparison of the mRNA levels for the indicated genes in the colon (A) and liver (B) of uninfected mice, and mice infected with the indicated Salmonella strains for 60 days, assessed by qPCR analysis. The data are presented as fold change relative to the housekeeping gene Hrpt. Statistical analysis was performed using non-parametric Mann-Whitney test. ***p value ≤ 0.001, **p≤ 0.01, and *p ≤ 0.05. (TIF) [file ppat.1005528.s004.tif]

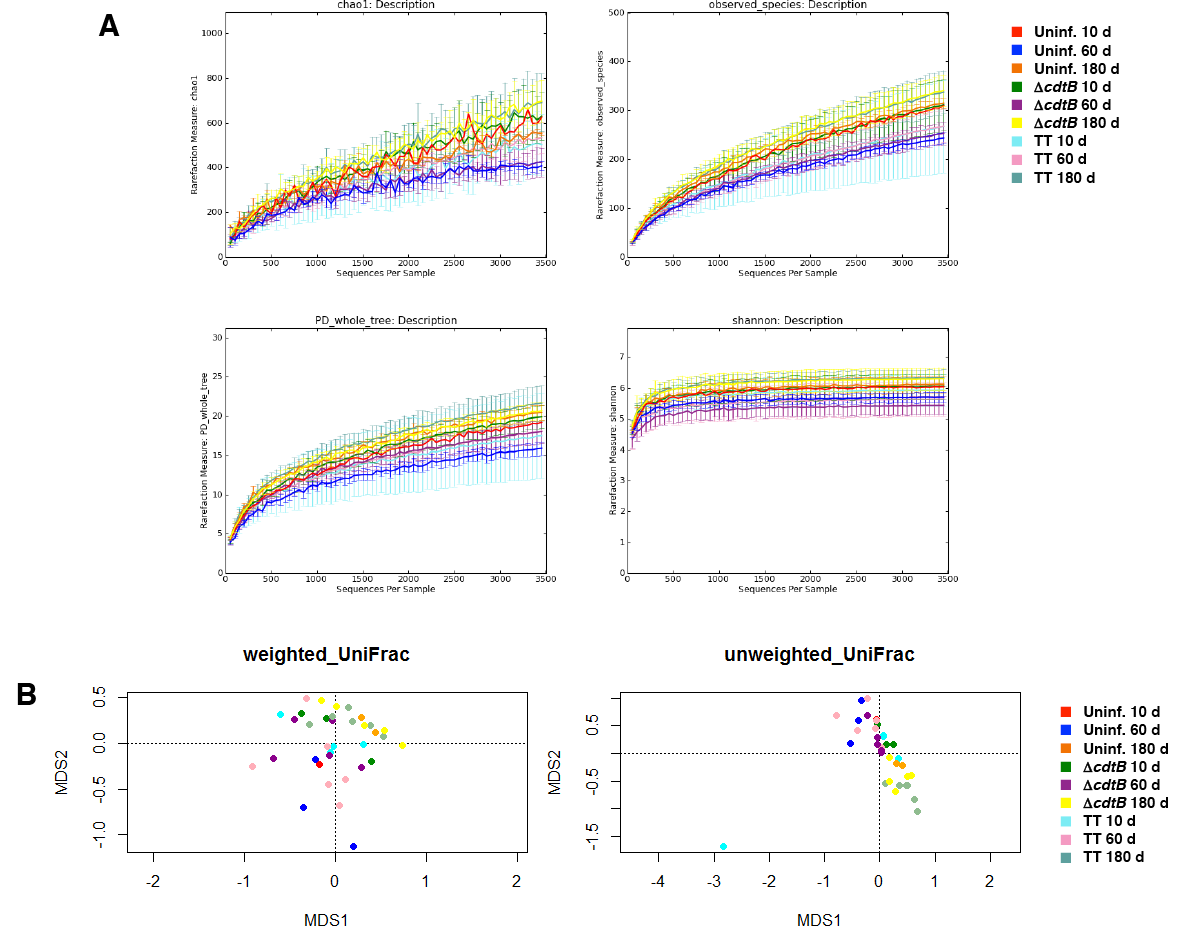

Supplement: S5 Fig — A. Alpha diversity rarefaction plots. Diversity was estimated by calculating the Chao1 measure of microbial richness, the observed number of OTUs, the Faith’s phylogenetic diversity index (PD whole tree), and the Shannon diversity index. B. Weighted and unweighted UniFrac Principal Component Analysis (PCoA). The two components explain 57.5 and 17.7% of the variance, respectively. Alpha and beta diversity of the intestinal microbiota were determined for uninfected mice and mice infected with the MC1-TT (TT) or MC1-ΔcdtB (ΔcdtB) strain at 10, 60 and 180 days post-infection. (TIF) [file ppat.1005528.s005.tif]

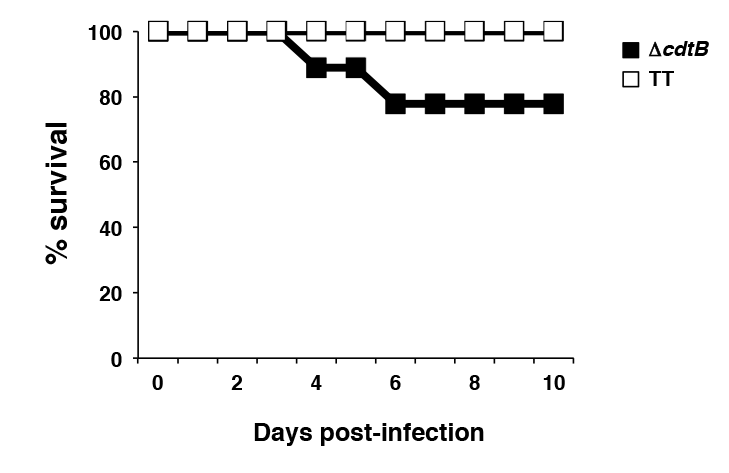

Supplement: S6 Fig — Five female C57BL/6 mice were infected orally with the S. Typhimurium MC71 strain carrying the inactive (∆cdtB) or functional toxin operon (TT) at an infection dose of 104 bacteria per mouse for 10 days. The Kaplan-Meier method was used to evaluate survival (95% confidence interval). (TIF) [file ppat.1005528.s006.tif]
